# Unusual Case of Severe Lactic Acidosis in a Liver Transplant Patient

**DOI:** 10.1155/2017/3704309

**Published:** 2017-12-17

**Authors:** Shweta Yemul Golhar, Michael S. Green, Stephen Guy

**Affiliations:** ^1^Department of Anesthesiology, Drexel University College of Medicine/Hahnemann University Hospital, Philadelphia, PA, USA; ^2^Department of Surgery, Drexel University College of Medicine/Hahnemann University Hospital, Philadelphia, PA, USA

## Abstract

Lactic acidosis is a standard indicator for oxygen debt and some other very significant causes. We describe a case of liver transplant patient presenting with vague abdominal pain and lactic acidosis without any liver dysfunction/failure/ischemia/rejection or sepsis. The imaging studies showed vague bowel edema and normal hepatic perfusion. The patient continued to deteriorate with rising lactic acidosis when a repeat CT abdomen eventually showed signs of lymphomatosis peritonei. Biopsy revealed the unusual diagnosis of posttransplant lymphoproliferative disorder. Immediate discontinuation of immunosuppression and initiation of chemotherapy led to clinical improvement. Our intention of presenting this case is to increase awareness of posttransplant lymphoma and propose lactic acidosis as not only an indicator of liver dysfunction or rejection but also an aid for diagnosis of this unusual but fatal and potentially curable condition.

## 1. Introduction

Lactic acidosis is a universally accepted marker for adequacy of perfusion. It is the end product of anaerobic metabolism and thus is commonly used as an indicator for oxygen debt. Lactic acid serves as a marker of mortality risk and a target for therapy [1, 2]. Lactic acidosis in a liver transplant patient holds profound implications. It is an indicator of early organ dysfunction, organ failure or rejection, infections, or severe sepsis and hence early detection can help initiate treatment and potentially alter the course of the condition [3]. However, there are some uncommon but very significant causes of severe lactic acidosis. We discuss a case of severe lactic acidosis in a liver transplant patient with an unusual cause and an ambiguous presentation.

## 2. Case Report

**Figure 1:**
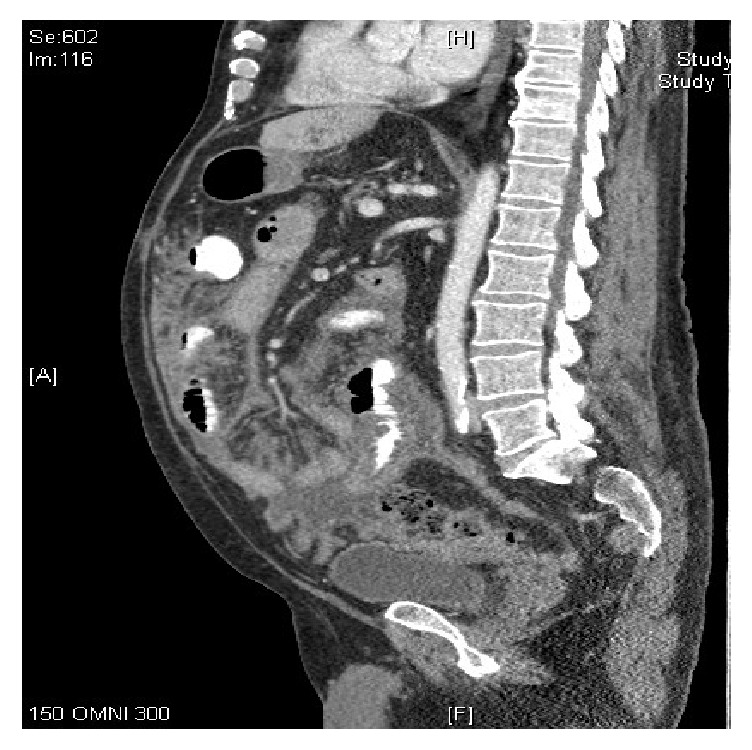
Diffuse nonspecific bowel edema.

A 68-year-old male with past medical history of orthoptic liver transplant secondary to NASH cirrhosis 4 years earlier and chronic kidney disease, presented with vague abdominal pain, weight loss, and fatigue. Mycophenolate and tacrolimus provided immunosuppression. Upon admission liver function tests (AST, ALT, and ALP), CBC, and tacrolimus level were all within normal limits. Additionally, the patient had an unremarkable RUQ ultrasound. He had some raised s. creatinine (1.58) compared to baseline of 1.28 due to poor oral intake. Patient was afebrile and vitals were stable, with no evidence of infection or sepsis. Hydration brought the creatinine down to baseline. A CT scan showed nonspecific bowel edema (Figure 1). Stool heme/WBC and culture, fungal culture, gram stain, AFB quantiferon, AFB culture, Yersinia, and urine culture were sent. LFTs and coagulation were still normal. Stool WBC was negative. Patient was still afebrile, without bandemia.

Colonoscopy showed an area at the distal ileum which was inflamed, granular, and necrotic, biopsy of which was consistent with nonspecific/ischemic ileitis. Antibiotics (ciprofloxacin and metronidazole) were started empirically due to ischemic changes in pathology. Later that day patient started complaining of increased abdominal pain concerning for bowel perforation; hence an immediate diagnostic laparoscopy was done which showed edematous bowel but no other significant abnormalities and ruled out ischemic bowel. Ascitic fluid gram stain was negative; ascitic fluid and peritoneal cultures were sent.

Intraoperatively, the patient suffered from severe metabolic acidosis with a pH of 7.08 and became hemodynamically unstable. The lactate levels remained high despite therapy and the pH further dropped to 6.9 despite fluid resuscitation. In ICU, sodium bicarbonate infusion and hemodialysis with bicarbonate dialysate was initiated to correct the acidosis and electrolyte abnormality. Vasoactive medications were initiated. Despite extensive efforts the lactate levels remained elevated. At this time, acute liver rejection, sepsis, portal/mesenteric arteriovenous thrombosis, or CMV infection were high on the differential diagnosis. However, liver enzymes remained normal and CT angiogram revealed patent vessels ruling out liver rejection/failure or any arteriovenous thrombosis. Mycophenolate was discontinued suspecting CMV ileitis. CMV PCR was sent. Urine analysis was normal. C. difficile toxin and EBV PCR laboratory evaluation was completed. Blood culture, sputum gram stain, CRE, stool WBC, and anaerobic blood culture were negative. PTLD was suspected. Tacrolimus was discontinued. Micafungin was added empirically considering fungemia as possibility. Pressors were tapered off slowly; LA peaked to 14 and then gradually came to 4–6 but never below that.

**Figure 2:**
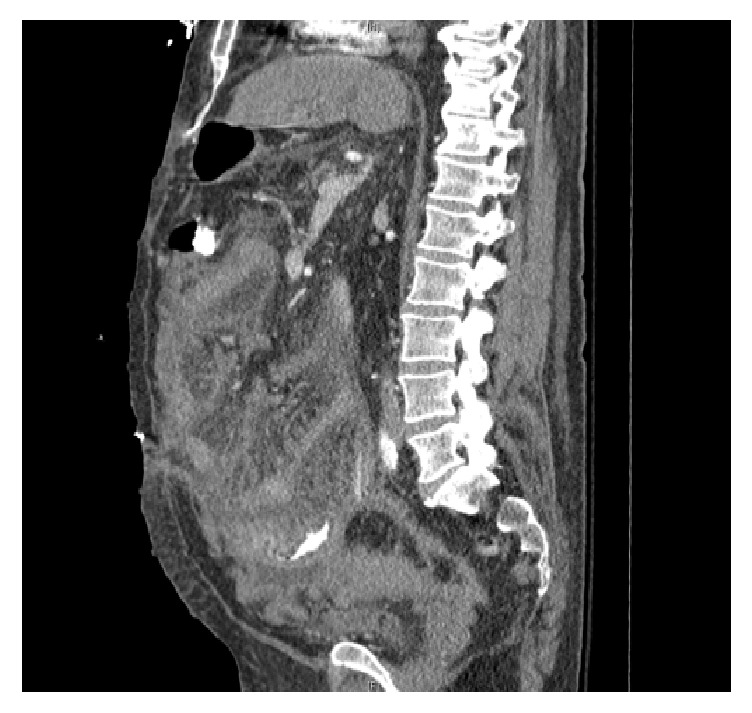
Mesenteric stranding and peritoneal thickening diagnostic for peritoneal lymphoma.

**Figure 3:**
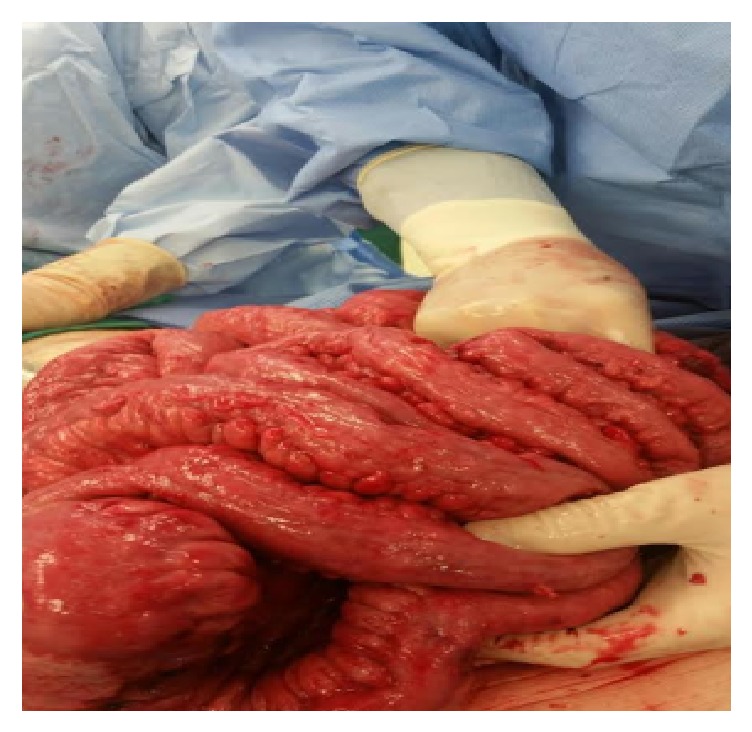
Variegated and pebble like peritoneum and bowel.

Repeat CT abdomen in a few days showed extensive bowel edema, mesenteric thickening, and stranding (Figure 2). Immediately, the patient was reexplored. At laparotomy, there was diffuse thickening of all bowel, mesentery, and peritoneum with several discrete lesions in the small bowel and mesentery. The surface was variegated and pebble like (Figure 3).

**Figure 4:**
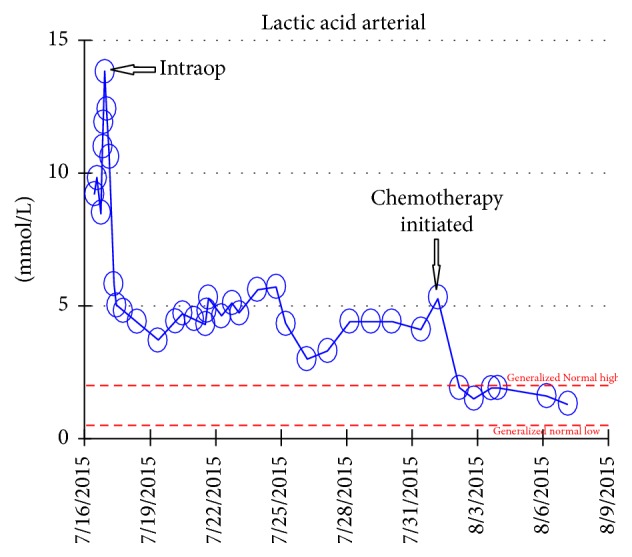
Lactic Acid Level timeline.

**Figure 5:**
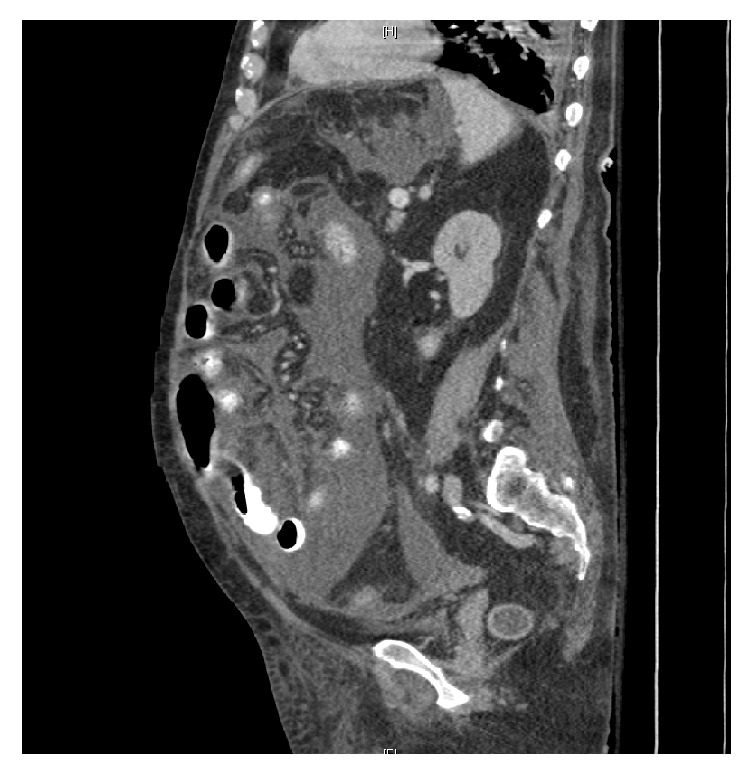
Resolution of the mesenteric and the peritoneal thickening after initiation of chemotherapy.

The peritoneal/omental and bowel biopsy was consistent with monomorphic Burkitt's lymphoma. Immediately, immunosuppression was held and chemotherapy was initiated. Rituximab and prednisone started initially and then Vincristine and cyclophosphamide. After the initiation of chemotherapy, the lactic acidosis started to resolve (Figure 4). Vasoactive medications were weaned and the patient was extubated. Repeat CT scan one week later showed tremendous improvement in the mesenteric thickening (Figure 5).

Following clinical improvement, unfortunately, the patient developed complications of chemotherapy associated pneumonia leading to sepsis and finally suffered pulmonary embolism. The patient deteriorated over next two weeks and care was withdrawn.

## 3. Discussion

Posttransplant lymphoproliferative disorder (PTLD) is a very grave complication of immunosuppression in transplant patients. PTLD is defined as the unrestrained growth of B cells in the face of T cell suppression. PTLD presents as both localized involvement or a more potent generalized disease. The UNOS database reports a 1.2% incidence in organ-transplant recipients [4]. The incidence is rising because of more potent immunosuppression and is higher in thoracic organs, hemopoietin stem cell, and intestine transplant and seems to increase with each passing year of transplant. Incidence of lymphoma in transplant recipients is 40 times higher than the general population [5]. The involvement can be nodal or extranodal with the most common sites being lymph nodes, liver, lung, kidney, and bone marrow [6]. Less common sites include the oral cavity and skin or subcutaneous lesions. Peritoneal involvement is extremely rare.

Most clinical symptoms are nonspecific and include fever, weight loss, lymphadenopathy, bowel obstruction, or mass effect. Extranodal disease is common but symptoms are very vague and presentation is very subtle. This can cause further delay in diagnosis which occurred in the patient presented [7]. There is a 6.8–24 times more association between EBV infection and lymphoproliferative disease [8]. However, EBV negative PTLD is more aggressive, has later presentation, and has poorer prognosis as was seen in our patient who was EBV negative. This explains the rapid fulminant spread within weeks and the aggressive nature of this disease. High lactic acid levels and a rapid fall after initiation of chemotherapy are indicators of high tumor load and remission, respectively.

Diagnosis of posttransplant lymphoma is made with the utilization of routine tests. No specific tests will yield a complete picture but many help contribute to the final diagnosis. CT scan, MRI, CXR, ultrasound, CSF if neurological involvement is suspected, bone marrow aspirate, PET scan, EBV viral load immunoglobulins, colonoscopy, biopsy of suspected lesion, cell markers like CD 20, immunohistochemistry, and LMP1 antigen all will provide valuable information. Our patient presented with normal liver function tests and negative EBV serology. The CT scan showed a very nonspecific edema of the bowel initially and subsequent biopsy was nondiagnostic. However, within the span of a week the repeat CT showed characteristic findings of peritoneal PTLD which includes mesenteric thickening and stranding. CT diagnosis of this condition especially without lymphadenopathy or any mass is challenging and unfortunately a late finding [9]. We believe that the disease was peritoneal to begin with and very rapidly spread to the peritoneum and that was the reason for negative biopsy at colonoscopy.

Mathur et al. reported a case of severe multiple organ failure in a patient following renal transplant. The patient presented with nonspecific symptoms and progressed to gradual failure of organs and severe lactic acidosis with liver failure. Reduction in immunosuppression did not alter the course and the patient expired. The diagnosis was made at autopsy which showed widely disseminated PTLD. This patient also showed very severe lactic acidosis without any response to treatment. In this case however, the liver failure also contributed to the high lactic acidosis, which was not the case in our patient [7].

Early diagnosis is the cornerstone of treatment as early reduction or discontinuation of immunosuppression allows the patient's natural immunity to recover and gain control over proliferating B cells. Other treatment modalities consist of chemotherapy, antiviral therapy, intravenous gamma globulin therapy, alpha interferon, radiation therapy, monoclonal antibodies, and cytotoxic T lymphocytes.

Lactic acidosis is an indicator of oxygen debt and is of 2 types. Type A is due to blood supply and demand becoming imbalanced. Type B is when the oxidative phosphorylation is hindered by toxins, drugs and inborn errors of metabolism. Lactic acidosis in a liver transplant patient is an ominous sign and is an indicator of liver dysfunction due to ischemia, congestion, liver cell failure due to early rejection, or infection/sepsis. Other unusual causes of lactic acidosis include malignancies, especially acute leukemia and high grade lymphoma. These generally have very poor prognosis. The cause of high lactic acidosis in lymphoma is mostly thought to be liver dysfunction, sepsis, and SIRS associated with these malignancies [10]. Overexpression of type II hexokinase or increased IGF-binding protein (IGFBP) activity has been implicated in the increased glycolysis in cancer cells [11, 12].

Weil and Afifi reviewed 20 case reports and found that a reduction in lactic acidosis in leukemia or lymphoma after initiation of chemotherapy was a good indicator for remission. In our case chemotherapy was initiated and lactic acid levels decreased. Severe lactic acidosis, even with maximal therapy, has a very high mortality of 60–90% and is an indicator for poor prognosis [13].

With increasing immunosuppression, use of more potent drugs, use of organs from extended criteria donors, and the increasing longevity of the transplant patients, the complications due to immunosuppression are occurring more frequently. The liver is a vital organ and unfortunately without any effective artificial replacement yet; hence diagnosis of early rejection or failure after liver transplant is extremely important. Use of lactic acidosis as an early indicator of liver dysfunction is very significant.

Our aim in reporting this case is to raise awareness of this rare but life threatening and potentially treatable cause of lactic acidosis in the liver transplant patients. PTLD should be included in any posttransplant patient with elevated lactic acid.
